# Supplementary material for: Epidemiologic Trends of Thalassemia, 2006–2018: A Nationwide Population-Based Study
Source: J Clin Med. 2022 Apr 20;11(9):2289. doi: 10.3390/jcm11092289 (PMC9104717; doi:10.3390/jcm11092289)
Supplement: Supplementary file 1 [file jcm-11-02289-s001.zip › jcm-1603993-supplementary.pdf]

Supplementary Data

Table of Contents

Supplementary Figures

Figure S1. The number of patients diagnosed with α-thalassemia or β-thalassemia…………2

Supplementary Tables

Table S1. Transfusion status in thalassemia and comorbidities………………………………..3

Table S2. Mean age at first marriage in Korea (2016 – 2020)…………………………………4

Table S3. Mean age of women at birth of first child in Korea (2016 – 2020)…………………5

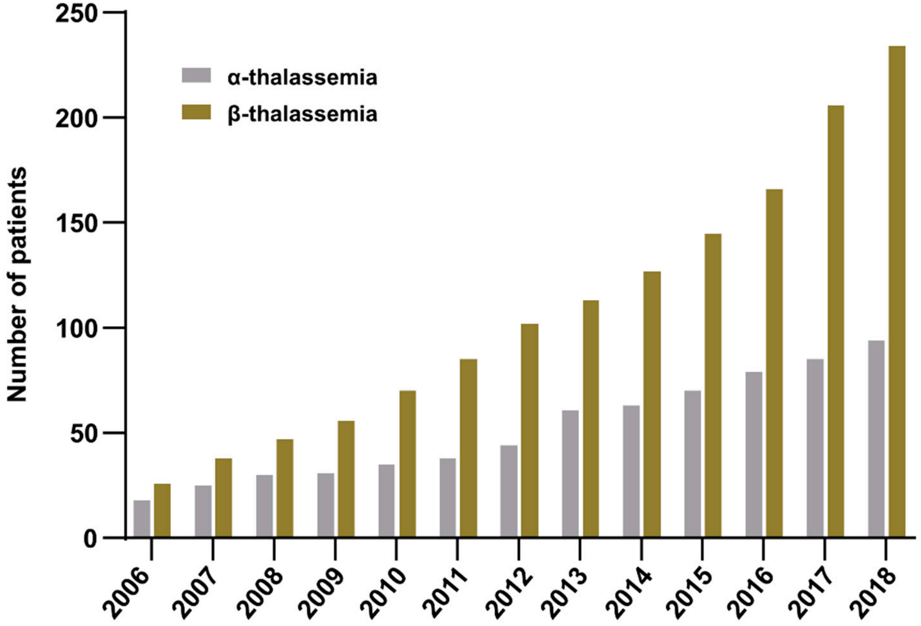

Figure S1. The number of patients diagnosed with α-thalassemia or β-thalassemia.

Table S1. Transfusion status in thalassemia and comorbidities.

Comorbidities	Transfusion Status	
	**Transfusion-Naïve Group**	**Transfusion Group**
Diabetes		
(-)	921	300
(+)	66	79
Hypertension		
(-)	824	236
(+)	163	143
Dyslipidemia		
(-)	888	318
(+)	99	61
Atrial fibrillation		
(-)	974	362
(+)	13	17
Myocardial infarction		
(-)	980	371
(+)	7	8
Stroke		
(-)	954	340
(+)	33	39
Heart failure		
(-)	962	353
(+)	25	26
End-stage renal disease		
(-)	957	344
(+)	30	35

Table S2. Mean age at first marriage in Korea (2016 – 2020) [23].

Regions	Male					Female				
	2016	2017	2018	2019	2020	2016	2017	2018	2019	2020
Nationwide	32.79	32.94	33.15	33.37	33.23	30.11	30.24	30.40	30.59	30.78
Seoul	33.20	33.36	33.51	33.72	33.61	31.01	31.20	31.32	31.55	31.60
Busan	33.04	33.23	33.32	33.54	33.39	30.63	30.74	30.91	31.04	31.08
Daegu	32.67	32.78	33.13	33.36	33.08	30.27	30.40	30.51	30.62	30.67
Incheon	32.67	32.80	33.03	33.25	32.96	30.06	30.19	30.39	30.59	30.70
Gwangju	32.94	33.08	33.28	33.48	33.38	30.14	30.30	30.40	30.65	30.68
Daejeon	32.47	32.38	32.67	32.90	33.00	29.95	29.97	30.27	30.45	30.61
Ulsan	32.35	32.44	32.79	32.78	32.74	29.74	29.93	30.13	30.25	30.33
Sejong	32.68	32.88	32.77	33.09	33.00	29.98	30.33	30.35	30.80	30.78
Gyeonggi	32.77	32.89	33.10	33.35	33.19	30.21	30.30	30.52	30.74	30.83
Gangwon	32.39	32.65	32.88	33.13	32.97	29.78	29.93	30.15	30.34	30.39
Chungbuk	32.41	32.27	32.57	32.72	32.63	29.56	29.67	29.65	29.98	30.15
Chungnam	32.27	32.63	32.87	33.05	32.85	29.27	29.59	29.70	29.91	29.95
Jeonbuk	32.75	32.77	33.41	33.67	33.54	29.65	29.72	30.04	30.34	30.49
Jeonnam	32.86	33.11	33.39	33.42	33.30	29.47	29.79	29.96	30.07	30.24
Gyeongbuk	32.56	32.75	32.90	33.30	33.01	29.75	29.82	30.13	30.41	30.38
Gyeongnam	32.54	32.77	32.90	33.20	33.06	29.91	30.04	30.20	30.47	30.52
Jeju	33.19	33.36	33.72	34.04	33.44	30.42	30.45	30.79	30.99	30.92

Table S3. Mean age of women at birth of first child in Korea (2016 – 2020) [23].

Regions	2016	2017	2018	2019	2020
Nationwide	31.37	31.62	31.89	32.16	32.30
Seoul	32.25	32.55	32.81	33.15	33.33
Busan	31.68	31.91	32.22	32.37	32.49
Daegu	31.41	31.54	31.71	31.94	32.08
Incheon	31.16	31.44	31.84	32.09	32.22
Gwangju	31.04	31.35	31.57	31.69	32.07
Daejeon	31.15	31.26	31.64	32.05	32.05
Ulsan	31.13	31.20	31.56	31.70	31.94
Sejong	31.51	31.75	32.06	32.35	32.61
Gyeonggi	31.46	31.73	32.01	32.24	32.39
Gangwon	30.63	30.78	30.96	31.28	31.32
Chungbuk	30.48	30.77	31.02	31.16	31.43
Chungnam	30.31	30.68	30.95	31.39	31.47
Jeonbuk	30.56	30.78	31.15	31.35	31.53
Jeonnam	30.55	30.70	31.00	31.30	31.25
Gyeongbuk	30.76	30.97	31.25	31.46	31.55
Gyeongnam	31.00	31.24	31.42	31.68	31.85
Jeju	30.93	31.25	31.56	31.87	31.83
